# Sex Differences in Time to Triage in the Emergency Department: A Retrospective Cohort Study

**DOI:** 10.5811/westjem.61906

**Published:** 2026-10-14

**Authors:** Siveen Hindiyeh, Abdallah Kurd, Avraham E. Alpert, Ariella Kovacs, Tehila Oren, Shaden Salameh

**Affiliations:** * Hadassah-Hebrew University Medical Center, Department of Emergency Medicine, Mount Scopus, Jerusalem, Israel; † The Hebrew University, Faculty of Medicine, Jerusalem, Israel; ‡ Hadassah-Hebrew University Medical Center, Department of Emergency Medicine, Ein Kerem, Jerusalem, Israel

## Abstract

**Introduction:**

Efficient triage in emergency departments (ED) is crucial for timely patient care, as evidence indicates that time to triage could affect treatment outcomes. Sex differences have been observed in different healthcare areas. The objective of this study was to determine whether sex differences exist in a patient’s time to triage and the magnitude of any observed differences at presentation to the ED of a Jerusalem hospital.

**Methods:**

This retrospective study used anonymized data from adult ED visits between January 1 to September 30, 2024, at an ED in Jerusalem. Our primary outcome measure was the mean time to triage, defined as the interval between documented patient arrival and the first encounter with a triage nurse. Our secondary outcome measures included the proportion of patients triaged within 15 minutes, stratified by sex, ED division, and Emergency Severity Index (ESI) level.

**Results:**

The study analyzed 30,447 adult ED visits over nine months. Mean time to triage was 10.9 minutes for women versus 10.3 minutes for men (absolute difference, 0.6 minutes; *P* < .001). Women were less likely to be triaged within 15 minutes (75.2% versus 76.9%; absolute difference, 1.7%; *P* < .001). Median time to triage was 6.0 minutes (interquartile range [IQR]) for men, 1.0–15.0 vs 7.0 minutes (IQR, 1.0–15.0) for women. Among patients with ESI levels 3–5, women had lower odds of triage within 15 minutes (odds ratio [OR], 0.78; 95% CI, 0.73–0.84; *P* < .001), while no significant sex differences were observed for ESI levels 1–2 (OR 1.30; 0.90–1.88; *P* = .17). Differences were most pronounced in ED triage to the ambulatory care area (absolute difference, 1.8%; OR, 0.95) and minimal in acute care (absolute difference, 0.5%; OR, 0.84), with no significant difference in the shock-trauma unit. Both sexes had similar triage rates within 60 minutes (99.2%).

**Conclusion:**

Although we found statistically significant sex differences in time to triage, the absolute difference of 0.6 minutes is not clinically meaningful. The near-universal triage within 60 minutes suggests that early triage prioritization differences do not translate into clinically significant delays. Within this ED, triage processes function similarly for men and women. Future research should evaluate downstream processes (eg, time to physician evaluation) to determine aspects of ED care that may demonstrate clinically important sex-based disparities.

## INTRODUCTION

Sex differences exist in multiple areas of healthcare. It is critical to examine whether there are differences between sexes in the timing of triage initiation in the emergency department (ED). For instance, the evaluations of door to room and door to clinician showed that men were more likely than women to be seen by the clinician and admitted to the hospital.^1^ Another study recognized that men have more severe initial symptoms, which could affect their faster processing and care provision.^2^

There are also documented differences between sexes in cardiovascular and stroke care, and for time to physician evaluation and proportion of hospital admission, as well as overdose treatment. There are significant cardiovascular health differences between men and women due to sex differences, including menopause and pregnancy, and gender differences, such as socialization, which contribute to the inequality risk.^3^ Coronary artery disease (CAD) is the leading cause of mortality in women.^4^ Women are more likely than men to develop nonobstructive CAD or microvascular dysfunction, where damage and thinning of the inner walls of blood vessels reduce blood flow and oxygen supply to the heart, causing chest pain.^5^ In general, perfusion problems, affecting blood flow to tissues and organs, are more common in women.

Recent data from a registry on ST-elevation myocardial infarction (STEMI) highlights that women experience significant delays in receiving reperfusion treatment, contributing to higher mortality rates in this population.^6^ Among more than 60,000 patients with STEMI, women have a significantly higher risk of in-hospital mortality.^7^ One study showed that by instituting a four-step protocol including ED catheterization lab activation, a STEMI handoff checklist, immediate transfer to the catheterization lab, and using the radial-first approach, the absolute sex difference in 30-day mortality could be reduced from 6.1% to 3.2% higher in women than men.^8^

Sex differences also exist in the treatment of acute stroke, as it was revealed that women manifest poorer functional recovery and lower quality of life after stroke compared to men.^9^ One study from Denmark showed that there was a median delay of 20 minutes from symptom onset to hospital arrival time for women compared to men, which may be one barrier to timely treatment.^10^ In a study based on data from the Houston metropolitan area, women were 9% less likely to be transported to a comprehensive stroke center even though they presented with a higher National Institutes of Health Stroke Scale score.^11^

In other areas of emergency medicine, delays in the ED assessment of poisonings (other than opioids) were documented in women.^12^ Research demonstrates that ED nurses perceive the middle-aged male patient to be more in need of urgent triage, admission to an intensive care unit (ICU) bed, and cardiac diagnosis than the age-matched female.^13^

The ED is the gateway to the hospital, and delays in identifying critical patients and starting care promptly can affect downstream outcomes. There is limited research that analyzes healthcare differences that occur in the initial processes in the ED. The objective of this study was to determine whether there are sex differences in the door-to-triage time of patients presenting to the ED of a Jerusalem hospital, and to examine the magnitude of any observed differences.

**Population Health Research Capsule**

What do we already know about this issue?

*Sex disparities exist in emergency department (ED) care, but differences in time to triage and their magnitude remain poorly defined.*

What was the research question?

*Do sex-based differences exist in time to triage and what is the magnitude of any observed differences?*

What was the major finding of the study?

*Women had a mean time to triage of 10.9 minutes vs 10.3 minutes for men (mean difference, 0.6 minutes; 95% CI, 0.30–0.90; P < .001).*

How does this improve population health?

*Identifying sex-related differences in time to triage may guide quality improvement and promote equitable emergency care.*

## METHODS

### Design and Settings

This was a quantitative retrospective study on sex differences in patients’ time-to-triage. The setting was an urban academic hospital ED in Jerusalem affiliated with the Hadassah Medical Center, with an annual census of 65,000. The methods of medical record review included choosing the criteria for case selection, identifying medical records, sampling the cases, forming the abstractions, and monitoring performance.^14^ We extracted anonymized patient demographics, site of triage (ambulatory care, acute care, shock-trauma unit), and Emergency Severity Index (ESI) category D from the institutional Business Intelligence electronic health record system. We excluded patients with incomplete or missing components.

### Study Participants

The study collected patient data from this ED on their visits throughout nine months, from January 1 to September 30, 2024. Our primary outcome measure was time to triage. The criteria for inclusion in the study were adult patients, patients admitted within the research period, and availability of information in the case studies (sex, age, ED division, ESI, and time to triage). Exclusion criteria were pregnant patients and women in advanced stages of pregnancy who were evaluated directly in the obstetric ED.

### Data Sources and Measurement

The 15-minute threshold was used as an operational benchmark for workflow assessment, consistent with ED performance metrics defined by the Ministry of Health, although this represents an administrative rather than clinical threshold. Time to triage was operationally defined as the interval between the patient’s recorded arrival in the ED and the initial encounter with the triage nurse. Our secondary outcomes were comparative results on time to triage in relation to sex, age, and ethnicity. Time to triage was subdivided among the ED’s three clinical divisions of the ED based on acuity and level of care: Ambulatory care for lower-acuity patients who can ambulate independently; acute care for patients with higher-acuity conditions requiring more intensive evaluation; and the shock-trauma unit for the most critically ill or injured patients requiring immediate resuscitation. All patients in the three divisions undergo standardized nurse-led triage using the ESI upon arrival, a five-point categorization where levels 1–2 are emergent/urgent, and levels 3–5 are lower acuity and depend on the number of resources anticipated. Arrival time was measured by the patient’s registration (administrative) arrival timestamp in the ED system.

### Statistical Analysis

We performed statistical analysis using the SPSS, version 27.0 (IBM Corp, Armonk, NY) and R statistical software version 3.5.0 (R Foundation for Statistical Computing, Vienna, Austria). Descriptive statistics are presented as means and standard deviations (SD) or as frequency (*n*) and percentage (%). Associations between time to triage, patient sex, ED clinical division, and ESI levels were assessed by simple linear and logistic regression, and were summarized by measures of associations, odds ratio (OR), and regression coefficient (β) together with 95% confidence intervals and *P* values. All significant variables at α = 0.2 were entered into a multivariable logistic regression model. A *P* value < .05 was considered statistically significant. All reported *P* values are two-tailed. The study was approved by the institutional review board (0035-25-HMO).

## RESULTS

### Descriptive Analysis

The study included a sample of 30,447 adult ED visits (15,584 men and 14,863 women). The analysis of the sample and descriptive data showed that women were older than men (mean age, 47.5 vs 43.7; *P* < .001). The data also showed that the mean time to triage for women was longer (10.9 minutes vs 10.3 minutes; *P* < .001) and that 76.9% of men (11,637/15,584) compared with 75.2% of women (10,952/14,863) underwent triage within 15 minutes (*P* < .001). Also, women were less represented in lower urgency levels (ESI 4–5: 5.6% of women vs 10.1% of men) (Table 1). The mean time to triage was 10.3 minutes for men and 10.9 minutes for women (Table 1). Median time to triage was 6 minutes for men and 7 minutes for women (Table 2). There was no meaningful difference between men and women in the proportion triaged within 60 minutes, with both groups at approximately 99.2% (Table 1).

These differences were observed across most age groups, although effect sizes remained small. When we further explored these findings according to the ESI level and the ED division, it was revealed that the time to triage in sex differences were significantly affected by ESI levels, with a notable difference in ESI 3–5 compared to ESI 1–2, where sex differences were less pronounced. Men were more likely than women (73.9% vs 72.1%; *P* < .001) to be triaged into the ambulatory care department. It is important to note that there is no universally established evidence-based threshold for clinically significant door-to-triage delay across all ED patients, because significance depends on acuity and clinical context. While some experts may consider delays greater than approximately five minutes potentially meaningful in patients with higher-acuity conditions, this remains largely based on expert opinion rather than validated evidence. Meanwhile, in most patients triaged within 15 minutes, men were more likely to be triaged within 15 minutes compared to women (76.9% vs 75.2%, Table 1). These findings complement the raw data and confirm that the observed sex differences in time to triage persist even after adjusting for ESI level, age, and ED division.

In data on median time-to-triage, women were triaged within 7 minutes, while men were triaged within 6 minutes (Table 2). Meanwhile, longer triage times were more frequently observed among men than women (Figure 1). The plot suggests that males tend to be triaged more speedily than females.

Overall, the results of the analysis show that men and women have similar but significant likelihood (OR, 0.93; 95% CI, 0.86–0.96; *P* < .01) of receiving triage within 15 minutes, independent of other variables (Table 3). In the shock-trauma unit, the results are significant (OR, 1.85; 95% CI, 1.35–2.60; *P* < .001). There were lower levels of age-related differences in younger age groups, where OR values approached 1.0. The association between sex and time to triage did not differ by ethnicity (Arabs vs Jews; interaction = .56; Table 3).

Figure 2 illustrates how odds of being triaged within 15 minutes varied by sex when stratified by key demographic and clinical subgroups. In terms of age group, women ≥ 85 years of age (OR, 0.79; *P* < .001) were most at risk for delays. In terms of ESI level 3–5 (OR; 0.78 for women [*P* < .001], the strongest effect was observed. Among patients with ESI levels 1–2, these differences were nonsignificant. In acute care (OR, 0.84), women showed more pronounced delay, while in ambulatory care (OR, 0.95), the delay was less pronounced.

## DISCUSSION

In this single-center retrospective study of approximately 30,000 ED visits, we identified statistically significant sex-based differences in time to triage across multiple patient subgroups. Across all age categories, females had lower odds of being triaged within the 15-minute benchmark compared to males. Despite reaching statistical significance, the absolute difference in mean time to triage between sexes was small—10.9 minutes for women compared to 10.3 minutes for men—suggesting that the observed differences are unlikely to represent clinically meaningful delays in most ED settings.

Importantly, triage within 60 minutes was nearly universal and did not differ between sexes, with approximately 99.2% of both women and men triaged within this timeframe. This finding indicates that the detected differences were limited to early triage prioritization rather than resulting in delayed overall access to triage. Together, these results suggest that while early ordering of triage may differ slightly by sex, such variation does not translate into prolonged delays in evaluation at the population level.

Several studies evaluating the factors affecting time to triage reported limited results. One study found that men and women experienced 6 and 7 minutes, respectively, in door-to-triage time.^15^ Another study showed an 11-minute difference in the same category, yet women and men were not differentiated within the sample.^16^ In door-to-room and door-to-healthcare time analyzed in another study, this measure differed from time-to-triage or door-to-triage time; significant sex-based differences were observed in length of stay and door-to-healthcare measures, where males were treated faster, and females remained longer in waiting areas.^12^ Another study examining ED triage found that female patients were less likely than male patients to be assigned the highest-acuity ESI categories, with disparities also observed by race and ethnicity.^17^ Compared with these studies, the current research remains unique in terms of the focus on sex differences in time-to-triage and their magnitude.

Subgroup analyses demonstrated that the magnitude of sex-based differences varied by age and care setting. The largest difference was observed among patients ≥ 85 years of age, in whom females had lower odds of being triaged within 15 minutes compared to males. However, this effect diminished progressively in younger age groups, with odds ratios approaching one. Differences were also more pronounced in acute care settings, while minimal differences were observed in ambulatory care. These findings suggest that higher-acuity or more protocol-driven environments may attenuate variation in early triage timing.

Women were more frequently classified into lower urgency categories than men, with 92.7% of women assigned to ESI levels 4–5 compared to 89.5% of men. Among patients triaged as ESI levels 3–5, females had lower odds of being triaged within 15 minutes than males. This pattern may reflect differences in presenting acuity, symptom profiles, or triage classification processes. Given the observational design of this study, it is not possible to determine whether these differences represent true variation in clinical presentation or differences in triage assessment.

Women were older than men in the study, which could have affected the results. Older women could have visited the ED more often, and their health issues might have been seen as less urgent. The bias toward patients might be mediated by the familiarity with some of the female patients and their age. Older adults tend to present to the ED more frequently, which might affect the intention of healthcare clinicians to hospitalize them.

Sex-based differences in early triage timing were consistent across ethnic groups, with similar patterns observed regardless of ethnicity. This consistency suggests that the observed differences were not confined to a specific demographic subgroup within the studied population and may reflect broader workflow or prioritization patterns within the ED rather than culturally or ethnically specific factors.

Taken together, these findings highlight the important distinction between statistical significance and clinical relevance in large datasets. While sex-based differences in time to triage were consistently detectable, their small absolute magnitude and the absence of downstream delay in triage within 60 minutes suggest limited clinical impact at the individual patient level. Nonetheless, even small and consistent differences may be relevant from a systems perspective, as they can reflect underlying patterns in triage workflows and prioritization mechanisms.

These results underscore the importance of cautious interpretation of statistically significant findings in large cohorts. The attenuation of differences in higher-acuity settings supports the potential role of standardized protocols in minimizing variability during triage. Rather than attributing observed differences solely to individual-level bias, future research should focus on system-level factors that may influence early triage prioritization, including ED crowding, staffing levels, clinician workload, and the use of decision-support tools.

Future studies should employ multicenter designs to assess the generalizability of these findings across different institutional settings and healthcare systems. Additional analyses incorporating downstream process and outcome measures—such as time to physician evaluation, diagnostic testing, disposition decisions, and clinical outcomes—are needed to determine whether early triage differences have meaningful clinical implications and to inform targeted, evidence-based system improvements where appropriate.

## LIMITATIONS

There are several limitations to this study. We used a retrospective design, which essentially limits causal inference. Even though statistical associations were determined between patient sex and time to triage, it is challenging to detect whether sex affects the delays or if there are unmeasured confounding variables that mediate this relationship. Nor was there a formal operational definition of gender, which does not include triage time differences for those with a different biological sex versus gender identity. Furthermore, the study was not inclusive of a full year of data.

The analysis was conducted at one hospital in Jerusalem, which limits the generalizability of the data. While the hospital serves a diverse urban population and includes both Jewish and Arab patients, other geographical regions could be under-represented. The sex of the triage nurse was not documented, which is a limitation; the study focused on differences in time to. triage by patient sex, while not accounting for nurse sex. However, it is worth noting that most nurses are female and of Jewish descent. Consequently, the lack of multimodal data decreases the chances of generating broader conclusions regarding the national or international trends in triage.

It is also important to note that while the ESI is a widely accepted triage tool, it is still subject to clinical judgment. Therefore, nurses can interpret ESI differently and acknowledge the urgency level based on their experience, biases, or subjective judgment, especially in cases with lower ESI levels. This triage approach may introduce unmeasured variability that is difficult to quantify. In addition, this research did not include inter-rater reliability assessments, which could have affected decision-making processes.

We did not collect patient-level clinical data beyond basic demographic information and triage classification. The mediating impact of data, including chief complaint, comorbid conditions, pain scores, and initial symptoms, was not included in the analysis, even though they are important in triage priorities. Therefore, it is unclear whether observed delays could be attributed to clinical presentation differences between men and women. Neither did we. report subgroup analysis for patients requiring an interpreter.

Finally, the study assessed the time to triage as the variable of interest. Delays in triage represent a single component of a broader aspect of care. For instance, the analysis of variables such as time to medical evaluation, time for diagnosis, intervention, or clinical outcomes could provide a wider picture of sex- or age-related differences.

## CONCLUSION

This single-center study of over 30,000 ED visits found no clinically meaningful sex-based difference in time to triage. Although statistically significant differences were detected, the absolute magnitude of these differences is too small to plausibly affect patient care. While the observed differences in time to triage appear to be clinically negligible, their impact on downstream care processes and patient outcomes remains uncertain and requires further evaluation. Future research should focus on downstream ED processes—including time to physician evaluation, diagnostic testing, disposition decisions, and clinical outcomes—to determine whether any aspect of emergency care demonstrates clinically meaningful sex-based disparities that warrant intervention. Additionally, multicenter studies are needed to determine whether the equitable triage processes observed here are generalizable to other institutions and healthcare systems.

## Figures and Tables

**Figure 1:**
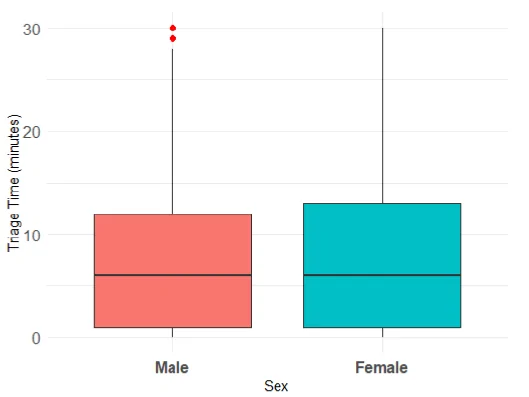
Boxplot visually comparing the distribution of time to triage time (in minutes) by sex among adult patients in an emergency department in Jerusalem.

**Figure 2:**
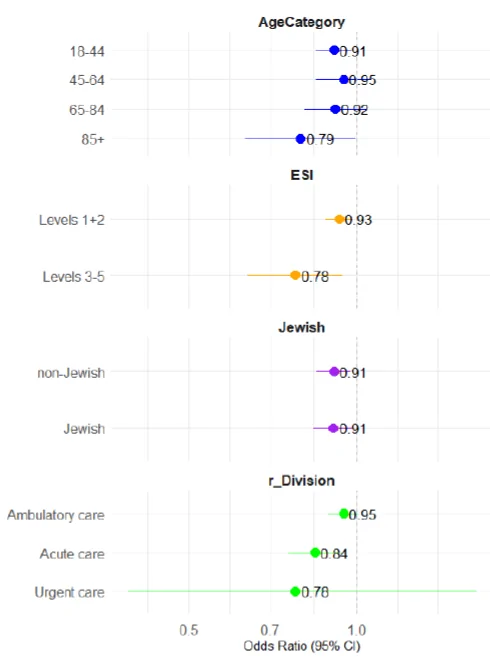
Odds ratios for timely triage (within 15 minutes) by sex across subgroups of age, emergency department division, and Emergency Severity Index level in a study of triage disparities. *ESI*, Emergency Severity Index.

**Table 1:** Descriptive characteristics of emergency department patients by sex in a study of sex-based differences in mean time to triage at a Jerusalem hospital.

Characteristic	Overall, *N* = 30,447^1^	Males, *n* = 15,584^1^	Females, *n* = 14,863^1^	*P* value^2^
ED Division				< .001
Ambulatory Care	22,207 (73%)	11,230 (72.1%)	10,977 (73.9%)	
Acute Care	7,839 (25.7%)	4,045 (26.0%)	3,794 (25.5%)	
Shock-Trauma Unit	401 (1.3%)	309 (2%)	92 (0.6%)	
ESI				< .001
1	217 (0.7%)	144 (0.9%)	73 (0.5%)	
2	2,473 (8.3%)	1,466 (9.6%)	1,007 (6.9%)	
3	24,813 (83.1%)	12,077 (79.4%)	12,736 (87.1%)	
4	2,061 (6.9%)	1,364 (9.0%)	697 (4.8%)	
5	282 (0.9%)	168 (1.1%)	114 (0.8%)	
Age (years)	45.6 (21.3)	43.7 (20.7)	47.5 (21.7)	< .001
Age Category				< .001
18–44	16,240 (53.3%)	8,663 (55.6%)	7,577 (51.0%)	
45–64	6,974 (22.9%)	3,648 (23.4%)	3,326 (22.4%)	
65–84	5,505 (18.1%)	2,647 (17.0%)	2,858 (19.2%)	
85+	1,728 (5.7%)	626 (4.0%)	1,102 (7.4%)	
Time to Triage	10.6 (13.4)	10.3 (13.6)	10.9 (13.0)	< .001
Time to Triage < 15 minutes	22,589 (76.1%)	11,637 (76.9%)	10,952 (75.2%)	< .001
Time to Triage < 60 minutes	29,463 (99.2%)	15,015 (99.2%)	14,448 (99.2%)	.77

1 N (%); Mean (SD).

2 Pearson Chi-squared test; Welch two-sample *t*-test

Descriptive statistics results for the main variables and demographic data of the sample.

**Table 2:** Comparison of median time to triage between male and female emergency department patients in a study of sex-based triage disparities.

Sex	Time to Triage (Median, Q1–Q3)	
	Males, *n* = 15,584^1^	Females, *n* = 14,863^1^
Time to triage	6.0 (1.0–15.0)	7.0 (1.0–15.0)
Unknown Triage Time	453	299

1 Median (25%–75%).

The data show the means and standard deviation results.

**Table 3:** Multivariable logistic regression analysis of categories associated with triage within 15 minutes in a study of sex-based triage disparities in a Jerusalem emergency department.

Categories	Males: Triage ≤ 15 minutes			Females: Triage ≤ 15 minutes		
	OR	CI	*P*	OR	CI	*P*
Age Category 45–64	1.05	0.98–1.12	.196	1.00	0.94–1.08	.90
65–84	1.02	0.95–1.10	.548	0.93	0.86–1.01	.06
[≥ 85]	0.85	0.76–0.96	.007	0.73	0.64–0.82	< .001
Jewish	1.02	0.96–1.07	.596	1.02	0.96–1.07	.56
Shock-trauma unit	1.85	1.35–2.60	< .001			
ESI (1 and 2)	1.30	1.17–1.45	< .001	1.22	1.09–1.36	< .001
Ambulatory care				0.77	0.71–0.82	< .001
Observations	29,647			29,647		

*ESI*, Emergency Severity Index; *OR*, odds ratio.
